# Flexible Atg1/ULK complex composition activates selective autophagy for phosphate starvation

**DOI:** 10.1186/s11658-024-00597-3

**Published:** 2024-06-04

**Authors:** Yijia Fangma, Zhong Chen, Yanrong Zheng

**Affiliations:** Key Laboratory of Neuropharmacology and Translational Medicine of Zhejiang Province, School of Pharmaceutical Sciences, Zhejiang Chinese Medical University, Hangzhou, Zhejiang China

**Keywords:** Atg1/ULK kinase complex, Phosphate starvation, Selective autophagy, Pexophagy

## Abstract

The molecular basis for bulk autophagy activation due to a deficiency in essential nutrients such as carbohydrates, amino acids, and nitrogen is well understood. Given autophagy functions to reduce surplus to compensate for scarcity, it theoretically possesses the capability to selectively degrade specific substrates to meet distinct metabolic demands. However, direct evidence is still lacking that substantiates the idea that autophagy selectively targets specific substrates (known as selective autophagy) to address particular nutritional needs. Recently, Gross et al. found that during phosphate starvation (P-S), rather than nitrogen starvation (N-S), yeasts selectively eliminate peroxisomes by dynamically altering the composition of the Atg1/ULK kinase complex (AKC) to adapt to P-S. This study elucidates how the metabolite sensor Pho81 flexibly interacts with AKC and guides selective autophagic clearance of peroxisomes during P-S, providing novel insights into the metabolic contribution of autophagy to special nutritional needs.

Autophagy is a finely orchestrated biological mechanism that reacts to changes in both external and internal metabolic requirements. The prevailing consensus asserts that autophagy is pivotal in supplying energy substrates during periods of nutritional restriction. By engulfing and breaking down proteins and organelles, autophagy enables the recycling of vital cellular constituents, including amino acids and phospholipids, back into the cytoplasm. This process fundamentally supports the survival needs of cells amidst starvation conditions. However, understanding the dietary requirements of cells is exceptionally complex. While yeast and plants rely on external elements such as nitrogen and phosphorus to sustain metabolism, mammals depend on external minerals and vitamins for optimal growth and development. Previous research suggests that the components present in various organelles vary significantly. For instance, ribosomes are rich in arginine and lysine, whereas peroxisomes, the Golgi, and the endoplasmic reticulum are abundant in phospholipids [1, 2]. Since autophagy functions to reduce surplus to compensate for scarcity, it theoretically possesses the capability to selectively degrade specific substrates to meet distinct metabolic demands.

Currently, the molecular basis for bulk autophagy activation due to a deficiency in essential nutrients such as carbohydrates, amino acids, and nitrogen is well understood. However, substantial experimental evidence is still needed to confirm the idea that autophagy selectively targets specific substrates (known as selective autophagy) to address particular nutritional needs. Recently, Gross et al. [3] found that during phosphate starvation (P-S) rather than nitrogen starvation (N-S), yeast selectively eliminate peroxisomes by dynamically altering the composition of the Atg1/ULK kinase complex (AKC) to adapt to P-S.

The AKC, composed of Atg1, Atg13, Atg17, Atg19, Atg31, and Atg11, plays a pivotal role in linking nutrient sensing with the induction of autophagic response. In the work by Gross et al. [3], it was discovered that during P-S but not N-S, yeast cells lacking Atg11, Atg13, or Atg17 maintained autophagy flux. However, yeast cells deficient in both Atg11 and Atg13 or Atg11 and Atg17 showed markedly reduced autophagy flux, indicating a functional compensation relationship between Atg11 and Atg13 or Atg17 under P-S conditions. Subsequently, through coimmunoprecipitation coupled with mass spectrometry, it was found that Pho81 was enriched in the AKC, but its presence was lost in the absence of Atg11. Further pulldown assay revealed the interaction between Pho81 and AKC mediated by Atg11 during P-S. To explore the contribution of Pho81 in AKC during P-S, high-resolution quantitative whole-cell proteomics was employed, and it was shown that both mitochondrial and peroxisomal proteins were accumulated in the absence of Pho81 or Pho81 and Atg11. After monitoring the turnover of mitochondrial and peroxisomal proteins and measuring the number of peroxisomes, they found that Pho81 specifically participated in pexophagy during P-S. Meanwhile, Pho81 was colocalized with Atg13 in regulating pexophagy during P-S. Moreover, P-S-induced pexophagy also required Atg1 and Atg11. These results indicate that Pho81 acts as an additional modulator of AKC in P-S-induced pexophagy.

To investigate the mechanism underlying Pho81 recruitment to AKC, the authors analyzed the activation state of autophagy-related proteins with whole-cell phosphoproteomics and found that TORC1, compared with N-S, was less phosphorylated and maintained its activity during P-S, leading to a higher level of phosphorylated Atg13. Interestingly, rapamycin incubation resulted in the rapid dephosphorylation of Atg13, promoting pexophagy during P-S independent of Pho81. These results indicated that, unlike N-S, TORC1 activity was not completely inhibited during P-S, allowing for the phosphorylation of Atg13. However, the activity of phosphorylated Atg13 was not robust, and therefore, AKC required Pho81 to compensate for the partial activation of Atg13 during P-S. The SPX domain of Pho81 is capable of phosphate ions (Pi) sensing and compensating for Atg13 inactivity. Following Pho81 recruitment to AKC, Pho81 further recruits Atg11 via the first loop of its ankyrin domain and promoted AKC-mediated phosphorylation of Atg11. The phosphorylation of Atg11 enhanced its interaction with the pexophagy receptor Atg36, thereby facilitating pexophagy [4, 5]. In summary, Gross et al. [3] have elucidated how the metabolite sensor Pho81 flexibly interacts with AKC and guides selective autophagic clearance of peroxisomes during P-S (Fig. 1).

**Fig. 1:**
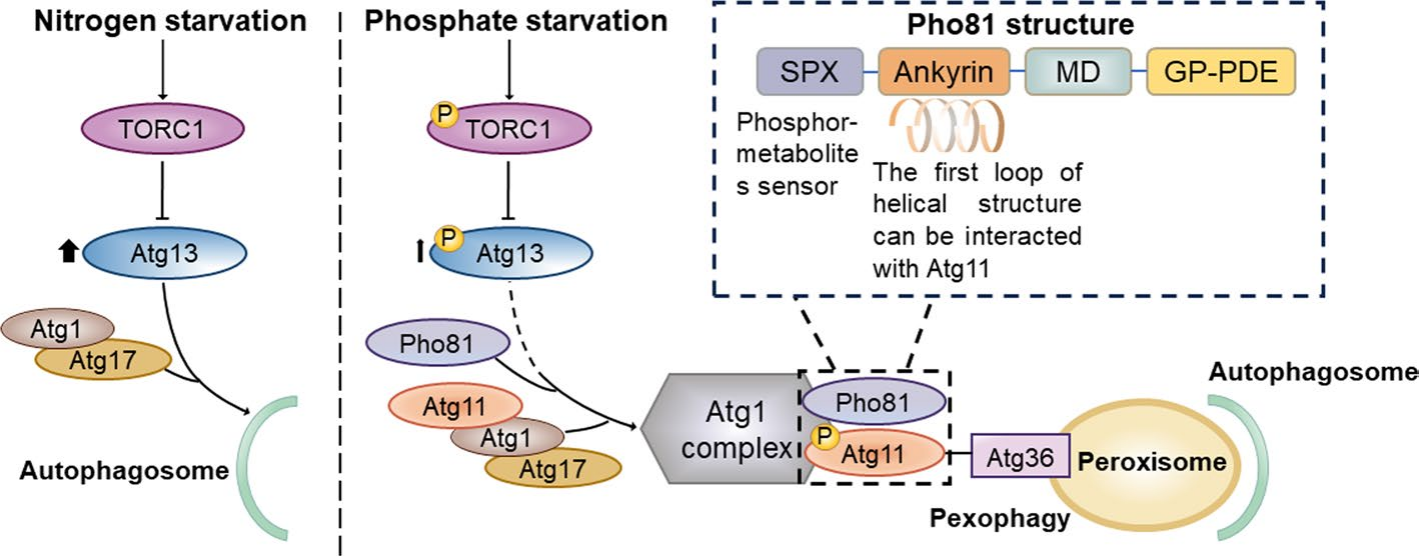
Flexible Atg1/ULK complex composition activates pexophagy for phosphate starvation. During nitrogen starvation, TORC1 is completely inhibited, resulting in the rapid dephosphorylation of Atg13. In contrast, upon phosphate starvation, TORC1 maintains some activity and Atg13 activity is relatively low; hence, Pho81 compensates for partially activated Atg13 by binding to Atg11, and Atg1 mediates phosphorylation of Atg11, which enhances its interaction with the pexophagy-receptor Atg36 and facilitates pexophagy. Mechanistically, Pho81 interacts with Atg11 via the first loop of its ankyrin domain. The SPX domain of Pho81, which is important for sensing phosphate ions (Pi) homeostasis, allows it to compensate for Atg13 inactivity and mediate Atg11-dependent pexophagy. *P* phosphorylated, *MD* minimal domain, *GP-PDE* glycerophosphodiester phosphodiesterase

Previous studies have shown that the composition of the AKC varies depending on the metabolic stressors, indicating its plasticity. For example, during N-S, inhibition of TORC1 activity results in Atg13 phosphorylation, facilitating the formation of Atg1–Atg13 and Atg17–Atg29–Atg31 complexes, which activate Atg1 and initiate autophagy [6]. In contrast, Atg11 is essential for glucose starvation, activating Atg1 by binding to Snf1 (an AMPK homolog) and promoting the formation of the Atg17–Atg29–Atg31 complex, along with recruiting Atg9 vesicles to the phagophore assembly site [4]. While these studies shed light on the molecular mechanisms of bulk autophagy activation under nutrient deficiency, the specific degradation of substrates triggered by different nutritional needs remains unclear. Notably, Gross et al. [3] proposed that P-S not only induces autophagy but also guides autophagosomes toward peroxisomes by dynamically modulating the composition of the AKC. However, the physiological implications of pexophagy remain unclear, which may potentially be linked to phosphate management during phosphate deficiency. In yeast and plants, peroxisomes play crucial roles in terpenoid synthesis, primarily existing in their phosphate-bound forms. Therefore, reducing the number of peroxisomes may limit the utilization of phosphate ions [7]. Additionally, peroxisomes are essential organelles involved in fatty acid β-oxidation [8], suggesting that pexophagy may also be associated with the synthesis of phosphate-free lipids and membrane lipid remodeling during phosphate starvation. Nonetheless, further clarification is needed to determine whether the abundance of other organelles, such as the endoplasmic reticulum, which is involved in phospholipid synthesis, has been altered.

Furthermore, autophagy plays a role in the degradation of metabolic enzymes. For instance, autophagy suppresses glycolysis by selectively degrading hexokinase 2, a crucial glycolytic enzyme, to reduce liver cancer progression [9]. Hence, it is imperative to investigate whether selective autophagy-induced degradation of metabolic enzymes acts as a strategy to cope with metabolic stress and elucidate the molecular mechanisms underlying this specificity. In mammals, despite the abundance of nutrient sources, deficiencies in certain trace elements can lead to various diseases and neurodevelopmental disorders such as depression, anxiety, schizophrenia, and autism [10]. Although several studies have shown a strong correlation between energetic insufficiency and selective autophagy, such as mitophagy [11] and lipophagy [12], the connection between specific nutrient or trace element deficiencies and autophagy remains unclear. Further research in related fields is needed to deepen our understanding of the pathophysiological mechanisms associated with developmental disorders and identify relevant drug targets.

## Conclusions

In summary, Gross et al. [3] provided novel insights into the metabolic contribution of autophagy to special nutritional needs and also established a framework for future research into the mechanisms and function of selective autophagy under different nutritional needs.

## Data Availability

Not applicable.

## Abbreviations

P-S: Phosphate starvation
N-S: Nitrogen starvation
AKC: Atg1/ULK kinase complex
